# Circadian disruption is associated with advanced cardio-kidney-metabolic syndrome in overweight and obese adults: evidence from the NHANES database

**DOI:** 10.1186/s12986-025-01072-2

**Published:** 2026-01-08

**Authors:** Qing Zhang, Tao Ye, Xiaolin Zhou, Bolin Li, Zhen Zhang

**Affiliations:** 1 Department of Cardiology, Affiliated Hospital of Southwest Jiaotong University, The Third People’s Hospital of Chengdu, Chengdu, 610014 Sichuan China; 2 Department of Cardiology, The First Affiliated Hospital of Xi’an Jiaotong University, 277 West Yanta Road, Xi’an, Shanxi 710061 China

**Keywords:** Overweight, Obesity, Circadian rhythm, Cardio-kidney-metabolic syndrome

## Abstract

**Background:**

Circadian disruption and obesity are increasingly recognized as key contributors to cardio-kidney-metabolic (CKM) syndrome. Yet, the extent to which circadian misalignment accelerates progression to advanced CKM stages in overweight and obese adults remains insufficiently defined.

**Methods:**

This study utilized both cross-sectional and longitudinal data from the National Health and Nutrition Examination Survey (NHANES) (2011–2014), focusing on overweight and obese adults aged 20 years and older. Circadian alignment was assessed by evaluating light-activity synchronization through phasor analysis, measuring phasor magnitude and acrophase, and the phasor magnitude was categorized into quartiles for subsequent statistical analysis. CKM stages (0–4) were used to represent progressive disease pathophysiology, with advanced stages (3 or 4) compared to nonadvanced stages (0, 1, or 2). Cross-sectional models were used to assess the association between circadian alignment and advanced CKM stages, and longitudinal survival models were applied to evaluate its associations with all-cause, cardiovascular, and premature mortality.

**Results:**

A total of 5,335 overweight and obese participants (median follow-up: 6.83 years) were included, representing approximately 111 million U.S. adults when survey weights were applied. After adjusting for age, sex, race, education, household income, smoking, alcohol consumption, hypertension, and diabetes, individuals with the lowest phasor magnitude—reflecting poor synchronization between light exposure and activity cycles—had significantly higher risks of advanced CKM stages (OR = 1.77, 95% CI: 1.17–2.68), all-cause mortality (OR = 2.12, 95% CI: 1.52–2.95), cardiovascular mortality (OR = 7.63, 95% CI: 2.82–20.63), and premature mortality (OR = 1.89, 95% CI: 1.07–3.36). Subgroup analyses further revealed that the associations between low phasor magnitude and both all-cause and cardiovascular mortality were stronger in older adults and men.

**Conclusions:**

Among overweight and obese individuals, lower phasor magnitude—indicating poor synchronization between light and activity cycles—was associated with higher risks of advanced CKM stages and increased mortality. These findings suggest that quantifying circadian disruption is important, and that interventions to enhance circadian alignment may help reduce CKM-related risks.

**Graphical Abstract:**

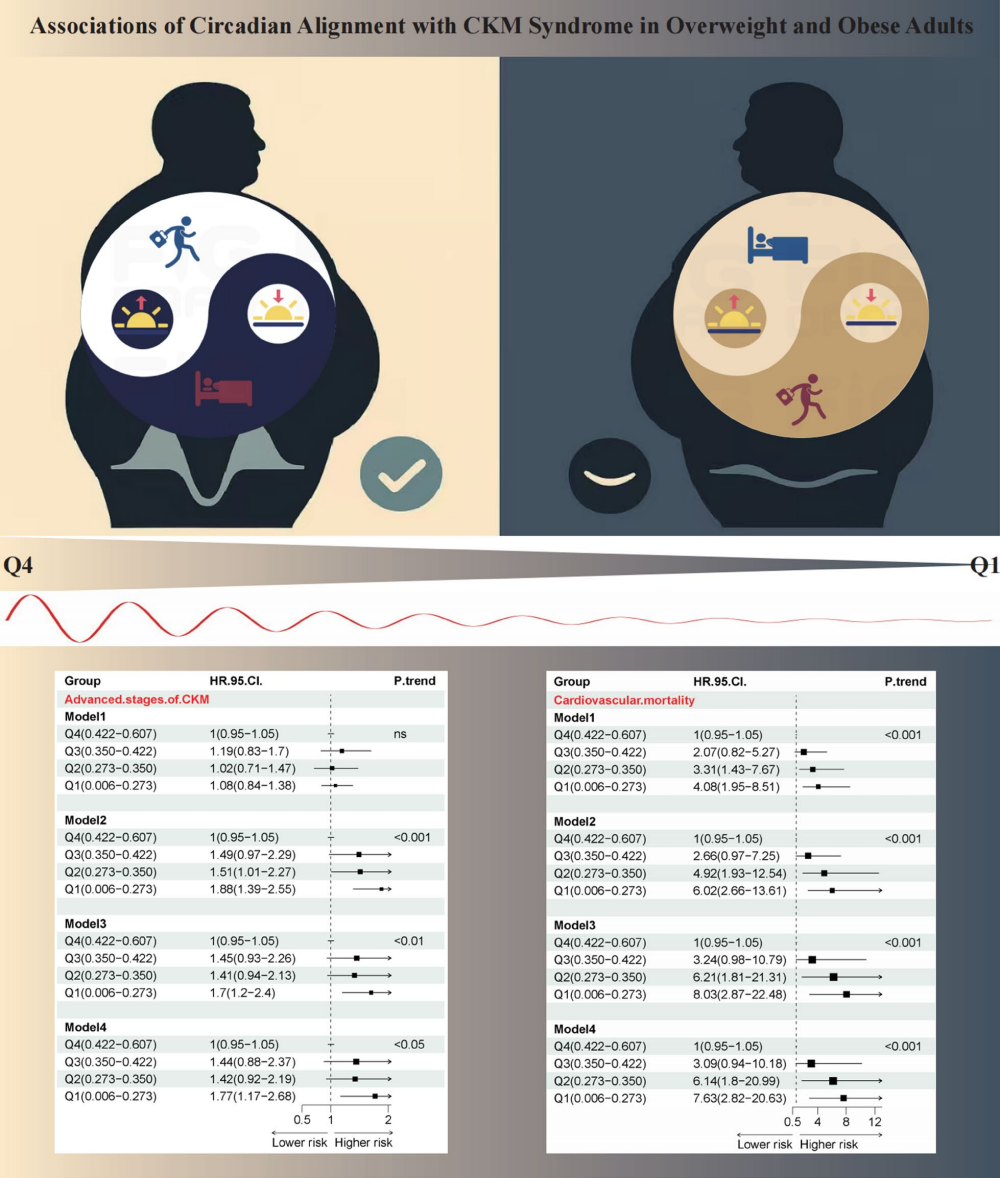

**Supplementary Information:**

The online version contains supplementary material available at 10.1186/s12986-025-01072-2.

## Background

Growing evidence of the interconnected nature of metabolic risk factors, chronic kidney disease (CKD), and the cardiovascular system has driven the emergence and conceptualization of cardio-kidney-metabolic (CKM) syndrome [1]. Rather than a simple coexistence of three conditions, CKM syndrome embodies a complex, bidirectional interplay among the cardiovascular, renal, and metabolic systems, which accelerates disease progression and increases mortality. The high global burden of CKM syndrome is evident: in the U.S., over a quarter of adults have at least one component of the cardio-kidney-metabolic triad, and internationally, the co-prevalence of these comorbidities reaches approximately 25–30%, emphasizing the need for integrated, cross-system approaches to prevention and care [2]. CKM syndrome is classified into five progressive stages: Stage 0 denotes the absence of identifiable CKM risk factors; Stage 1 reflects the presence of early risk indicators such as excess adiposity; Stage 2 is defined by manifest cardiometabolic risk factors and/or chronic kidney disease; Stage 3 involves subclinical cardiovascular disease coexisting with renal and metabolic abnormalities; and stage 4 represents overt clinical cardiovascular disease, with or without concurrent CKD. Advanced CKM syndrome encompasses stages 3 and 4 of the CKM continuum, characterized by subclinical or clinical cardiovascular disease, progressive kidney impairment, and persistent metabolic dysfunction. Although advanced CKM syndrome affects only 12.8% of U.S. adults, it carries a disproportionately elevated risk of adverse outcomes, with cardiovascular mortality reaching a 15-year cumulative incidence of 23.9% [3, 4]. Accordingly, delineating advanced CKM syndrome holds significant clinical and public health implications, informing strategies to mitigate patient risks and lessen the strain on healthcare systems.

Multiple modifiable and non-modifiable factors contribute to CKM health decline, including hypertension, insulin resistance, dyslipidemia, sedentary behavior, and genetic predisposition. Central to these is obesity, which serves as both the starting point and the key accelerator of CKM syndrome [5]. Even during the era when the concept of cardiorenal syndrome was predominant, obesity had already been recognized as a significant contributor to both cardiovascular and kidney-related diseases [6]. The incorporation of obesity-centered metabolic syndrome into the CKM framework has further underscored the pivotal role of obesity in the development and progression of CKM. With global projections indicating an increase in adult obesity from 43.4% in 2021 to 57.4% by 2050, addressing the risks and outcomes of CKM in obese populations has become an increasingly urgent public health priority [7].

Circadian rhythms are intrinsic 24-hour cycles that govern a wide array of physiological and behavioral processes, including sleep–wake cycles, hormone secretion, energy metabolism, and cardiovascular function. These rhythms are regulated by a central pacemaker located in the suprachiasmatic nucleus of the hypothalamus, which synchronizes peripheral clocks through neuronal and hormonal signals [8, 9]. Circadian rhythms have become a major research focus due to their fundamental role in regulating sleep, metabolism, cardiovascular function, and immune responses. Advances in chronobiology have revealed that disruption of these rhythms—caused by shift work, irregular sleep, or light pollution—is linked to increased risks of obesity, diabetes, cardiovascular disease, and cancer [10–13].

Obesity and circadian rhythm disruption have each been independently shown to play significant roles in the development of cardiovascular and kidney-related diseases [14, 15]. However, no studies to date have specifically examined the impact of circadian disruption on the risk and outcomes of CKM syndrome in overweight and obese populations.

## Materials and methods

### Data source and study population

This study utilized publicly available data from the National Health and Nutrition Examination Survey (NHANES), a population-based survey employing stratified, multistage probability sampling to evaluate health and nutritional status among U.S. residents. Institutional review board approval for NHANES was granted by the National Center for Health Statistics, and all data used in this secondary analysis were de-identified. Reporting followed the STROBE guidelines for cross-sectional studies.

Participants were drawn from the 2011–2012 and 2013–2014 NHANES cycles. Overweight and obesity were defined as BMI ≥ 25 kg/m²; Exclusion criteria included age < 20 years and missing data on CKM syndrome, anthropometric measurements, survival status, or device-based light/activity assessment. The final sample comprised 5,335 overweight or obese adults, representing a weighted population estimate of 110,542,951, with a median follow-up of 6.83 years (Figure S1).

### Phasor analysis

In this study, light exposure and physical activity data were collected from NHANES participants using the GT3X + ActiGraph accelerometer. Light exposure were recorded using the PAXLXSH variable (in lux), while physical activity intensity was quantified through the PAXMTSH variable. To align the light and activity signals temporally, the light data were logarithmically transformed, and a saturation threshold of 10,000 lux was applied to account for the nonlinear circadian response, while the physical activity data were similarly transformed. They were segmented into consecutive 24-hour periods, and the signals were multiplied point-by-point and normalized to generate a correlation coefficient time series. By circularly shifting one signal relative to the other, a behavioral synchronization correlation function was computed, and its 24-hour frequency component was extracted.

**Fig. 1:**
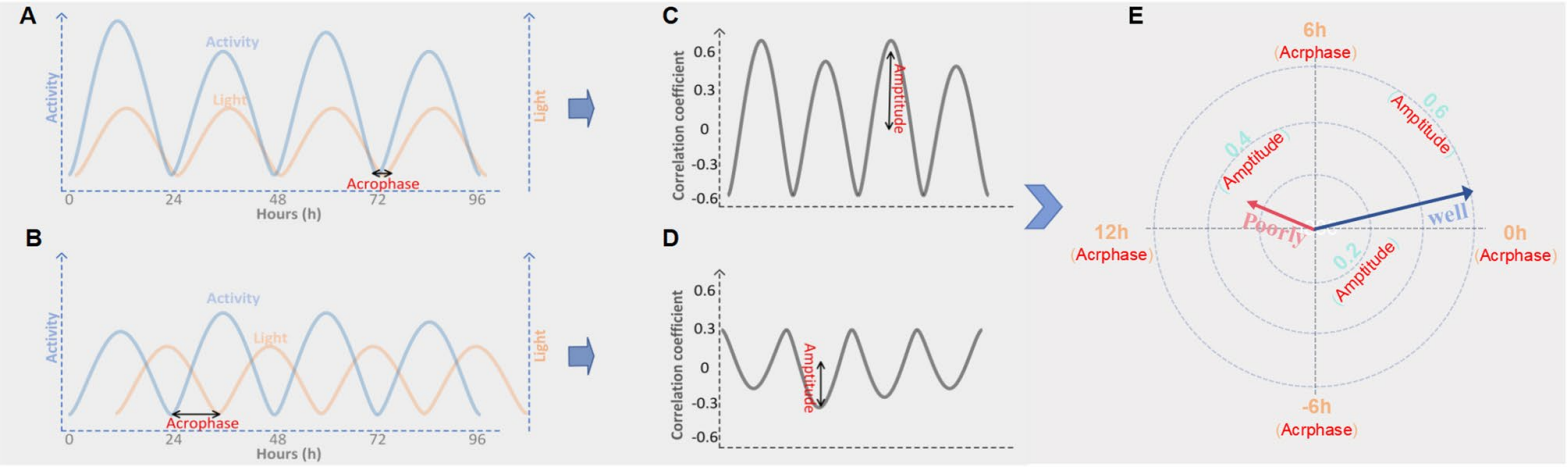
Phasor analysis of circadian synchronization. (**A**-**B**) show multi-day records of light exposure (orange) and activity (blue) from two participants, with the time of minimum activity (nadir) marked. In (**A**), activity and light rhythms are well aligned, whereas (**B**) shows a mismatch. (**C**-**D**) display correlation functions for behavioral synchronization, calculated by shifting one signal against the other within each 24-hour cycle. The size of the 24-hour component indicates how strongly the two rhythms are synchronized. (**C**) demonstrates strong and regular coupling, while (**D**) shows weaker and inconsistent coupling. (**E**) is a phasor plot that summarizes overall synchronization. The vector length represents the stability and strength of alignment, and the angle shows the phase of activity relative to light. The “well” vector corresponds to (**A**, **C**), and the “poorly” vector corresponds to (**B**, **D**)

Phasor analysis was performed to quantify the alignment between the light–dark cycle and physical activity rhythms. Phasor acrophase reflects the temporal relationship between the peaks or troughs of activity and light (Fig. 1A and B). A positive value indicates that activity lags behind light exposure (evening preference), while a negative value indicates that activity precedes light exposure (morning preference). Values near zero denote close alignment with the external light–dark cycle, suggesting synchrony between light and activity. Additionally, the phasor magnitude, which reflects the strength and regularity of the light–activity coupling, was derived from the 24-hour frequency component (Fig. 1C and D). A larger phasor magnitude indicates stronger and more regular synchronization between light exposure and activity, while a smaller magnitude suggests weaker or less consistent coupling. Phasor vectors were visualized in polar coordinates, where the vector length represents the synchronization strength, and the angle indicates the phase relationship between light and activity(Fig. 1E) [16, 17].

### Definition CKM syndrome

CKM syndrome stages (0–4) were categorized according to modified criteria based on Aggarwal et al. [18], adapted for NHANES data fields: Stage 0: Waist circumference below 88 cm (female) or 102 cm (male), with no higher stage criteria met. Stage 1: BMI ≥ 25 kg/m², WC ≥ 88 cm (female) or ≥ 102 cm (male), or prediabetes (fasting glucose 100–124 mg/dL, HbA1c 5.7–6.4%, or antidiabetic therapy). Stage 2: Presence of metabolic risk factors (triglycerides ≥ 135 mg/dL, hypertension, diabetes, or metabolic syndrome), or moderate/high-risk CKD [19]. Stage 3: Very high-risk CKD or elevated 10-year CVD risk. Stage 4: History of CVD [20]. CKM syndrome was further classified as advanced (stages 3–4) or non-advanced (stages 0–2).

### Outcomes

Primary outcomes included all-cause mortality, cardiovascular mortality (ICD-10 I00–I99), and premature death (before age 70), ascertained via linked national death records.

### Covariates

Potential confounders were selected a priori, including age, sex, race (Hispanic, non-Hispanic Black, non-Hispanic White, other), smoking status, alcohol consumption, educational attainment, household income, hypertension, and diabetes. All covariate data were self-reported and categorized according to NHANES standards.

### Statistical analysis

NHANES sampling weights (WTINT2YR) and appropriate design variables were applied in all analyses to ensure nationally representative estimates. Quartiles grouped phasor magnitude and angle to characterize the research population. Descriptive statistics for continuous variables are presented as weighted means ± SE or medians with 95% CIs; categorical variables as weighted proportions. Between-group comparisons were conducted using the Wald F test, Kruskal–Wallis test, or Rao–Scott χ² test.

Weighted logistic regression models were employed to investigate the association between phasor parameters and the risk of advanced CKM syndrome. To assess relationships with mortality outcomes, Cox proportional hazards regression was performed. All models were fitted with sequential adjustment: Model 1 included no covariates (unadjusted); Model 2 incorporated demographic variables; Model 3 further adjusted for lifestyle factors; and Model 4 additionally included clinical covariates. Restricted cubic splines tested for potential nonlinear associations, controlling for all covariates in Model 4.

Analyses were performed using R version 4.4.1 (R Foundation for Statistical Computing, Vienna, Austria).

## Results

### Baseline characteristics of participants

Descriptive characteristics of the study population, stratified by phasor magnitude quartiles, are summarized in Table 1. 5335 participants, corresponding to a nationally representative estimate of 110,542,951 individuals based on NHANES survey weights, were included in the final analysis with a follow-up duration of 6.83 years (Weighted data are presented in supplementary Table 1). The mean age of the cohort was 51.8 years; 49.3% of the participants were male, and 41.8% were non-Hispanic White. The prevalence of current smoking and alcohol consumption was 17.5% and 61.7%, respectively. Furthermore, 50.3% of individuals had hypertension, 22.8% had diabetes, and 18.6% met criteria for advanced CKM syndrome. Participants were categorized into quartiles based on phasor magnitude values: Q1 (0.20 [0.06]), Q2 (0.31 [0.02]), Q3 (0.39 [0.02]), and Q4 (0.48 [0.04]). The mean ages in Q1 through Q4 were 50.0, 51.0, 53.3, and 52.8 years, respectively. Corresponding median body mass index(BMI) values were 32.9, 32.2, 31.9, and 31.3 kg/m².

**Table 1:** Baseline demographic and clinical characteristics of the study population

Characteristic	Overall *N* = 5,335^1^	1 *N* = 1,334^1^	2 *N* = 1,334^1^	3 *N* = 1,333^1^	4 *N* = 1,334^1^	*p*-value^2^
**Magnitude**	0.34 (0.11)	0.20 (0.06)	0.31 (0.02)	0.39 (0.02)	0.48 (0.04)	< 0.001
**Age**	51.8 (16.5)	50.0 (16.9)	51.0 (16.7)	53.3 (16.5)	52.8 (15.7)	< 0.001
**Gender = male**	2631 (49.3)	696 (52.2)	647 (48.5)	626 (47.0)	662 (49.6)	0.052
**Race**						< 0.001
Mexican American	725 (13.6)	110 (8.2)	163 (12.2)	199 (14.9)	253 (19.0)	
Hispanic	545 (10.2)	107 (8.0)	136 (10.2)	154 (11.6)	148 (11.1)	
Non-Hispanic White	2231 (41.8)	499 (37.4)	511 (38.3)	577 (43.3)	644 (48.3)	
Non-Hispanic Black	1335 (25.0)	465 (34.9)	396 (29.7)	285 (21.4)	189 (14.2)	
Other	499 (9.4)	153 (11.5)	128 (9.6)	118 (8.9)	100 (7.5)	
**Education**						< 0.001
College graduate or above	1197 (22.4)	327 (24.5)	358 (26.8)	289 (21.7)	223 (16.7)	
High school graduate	1224 (23.0)	293 (22.0)	285 (21.4)	297 (22.3)	349 (26.2)	
Less_than_high_school	1233 (23.1)	239 (17.9)	268 (20.1)	339 (25.4)	387 (29.0)	
Some college	1679 (31.5)	474 (35.6)	423 (31.7)	408 (30.6)	374 (28.1)	
**Household.income**						0.026
< 20,000	1177 (22.9)	308 (23.8)	295 (23.0)	291 (22.7)	283 (22.1)	
20,000–44,900	1767 (34.4)	438 (33.9)	416 (32.4)	434 (33.9)	479 (37.4)	
45,000–74,900	913 (17.8)	214 (16.6)	215 (16.8)	253 (19.8)	231 (18.0)	
75,000+	1280 (24.9)	333 (25.8)	356 (27.8)	303 (23.7)	288 (22.5)	
**BMI**	32.1 (6.27)	32.9 (7.23)	32.2 (6.15)	31.9(6.10)	31.3 (5.37)	< 0.001
**Smoking**	936 (17.5)	282 (21.1)	242 (18.1)	196 (14.7)	216 (16.2)	< 0.001
**Alcoh.week**						< 0.001
< 1/week	1881 (35.3)	479 (35.9)	488 (36.6)	471 (35.3)	443 (33.2)	
1/day above	193 (3.6)	30 (2.2)	36 (2.7)	50 (3.8)	77 (5.8)	
1/week to < 1/day	1216 (22.8)	302 (22.6)	294 (22.0)	313 (23.5)	307 (23.0)	
NO	2045 (38.3)	523 (39.2)	516 (38.7)	499 (37.4)	507 (38.0)	
**Hypertension**	2684 (50.3)	679 (50.9)	661 (49.6)	708 (53.1)	636 (47.7)	0.039
**Diabetes**	1217 (22.8)	330 (24.7)	306 (22.9)	299 (22.4)	282 (21.1)	0.168
**adv.CKM_stage**	856 (18.6)	211 (18.8)	214 (18.5)	226 (19.3)	205 (17.7)	0.792
**Albuminuria**						0.085
A1	4038 (87.8)	959 (85.7)	1037 (89.8)	1021 (87.3)	1021 (88.2)	
A2	471 (10.2)	130 (11.6)	97 (8.4)	128 (10.9)	116 (10.0)	
A3	92 (2.0)	30 (2.7)	21 (1.8)	21 (1.8)	20 (1.7)	
**eGFR**						0.001
G1	2718 (59.1)	654 (58.4)	660 (57.1)	661 (56.5)	743 (64.2)	
G2	1518 (33.0)	366 (32.7)	407 (35.2)	405 (34.6)	340 (29.4)	
G3a	228 (5.0)	53 (4.7)	51 (4.4)	76 (6.5)	48 (4.1)	
G3b	100 (2.2)	29 (2.6)	30 (2.6)	22 (1.9)	19 (1.6)	
G4	30 (0.7)	13 (1.2)	6 (0.5)	5 (0.4)	6 (0.5)	
G5	8 (0.2)	5 (0.4)	1 (0.1)	1 (0.1)	1 (0.1)	
**CKD.risk**						< 0.001
low	3792 (82.4)	900 (80.4)	978 (84.7)	942 (80.5)	972 (84.0)	
mod	560 (12.2)	140 (12.5)	111 (9.6)	170 (14.5)	139 (12.0)	
hig	164 (3.6)	49 (4.4)	45 (3.9)	44 (3.8)	26 (2.2)	
vhig	86 (1.9)	31 (2.8)	21 (1.8)	14 (1.2)	20 (1.7)	
**Heart failure**	211 (4.0)	66 (4.9)	50 (3.7)	54 (4.1)	41 (3.1)	0.095

^1^ Median (Q1.Q4); n (%)

^2^ Design-based KruskalWallis test; Pearson’s X^2: Rao & Scott adjustment

### Association between circadian alignment and advanced CKM risk

**Fig. 2:**
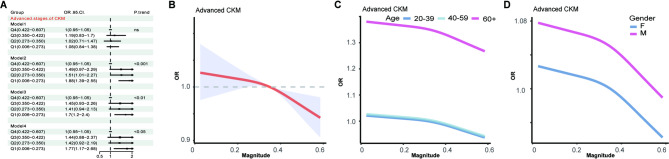
Association between phasor magnitude and odds of advanced cardio-kidney-metabolic (CKM) syndrome in overweight and obese adults. Panels **A** shows forest plots of odds ratios (ORs) with 95% confidence interval (CI) across quartiles of phasor magnitude for advanced CKM from four multivariable logistic regression models (Model 1: unadjusted. Model 2: adjusted for age, sex, race, education level, and household income. Model 3: Model 2 + smoking status + weekly alcohol consumption. Model 4: Model 3 + hypertension and diabetes mellitus.). Panels **B** depict restricted cubic spline curves of ORs (solid lines) and 95% CIs (shaded areas) for advanced CKM as a continuous function of phasor magnitude. Panels **C** and **D** present age-stratified (20–39, 40–59, ≥ 60 years) and gender-stratified (female, male) OR curves for advanced CKM

We focused on examining whether circadian rhythm disruption is associated with the risk of advanced CKM syndrome and find that lower phasor magnitude was associated with an increased risk of advanced CKM syndrome across all adjusted models (Fig. 2A). In Model 2, participants in the lowest quartile (Q1) had a significantly higher risk compared to Q4 (OR = 1.88, 95% CI: 1.39–2.55). This association remained significant in Model 3 and Model 4, with Q1 showing ORs of 1.70 (95% CI: 1.20–2.40) and 1.77 (95% CI: 1.17–2.68), respectively. Restricted cubic spline analysis demonstrated a significant inverse association between phasor magnitude and the risk of advanced CKM syndrome (Fig. 2B), indicating that lower phasor magnitude was associated with higher odds of advanced CKM. Age-stratified analysis showed that the inverse association between phasor magnitude and advanced CKM syndrome was observed across all age strata, with similar slopes. However, the overall risk was consistently higher among participants aged ≥ 60 years compared with younger groups (20–39 and 40–59 years)(Fig. 2C). Gender-stratified analysis revealed a higher risk gradient in males compared to females (Fig. 2D). However, acrophase quartiles were not significantly associated with the risk of advanced CKM stages (Figure S2).

### Association between circadian alignment and mortality

**Fig. 3:**
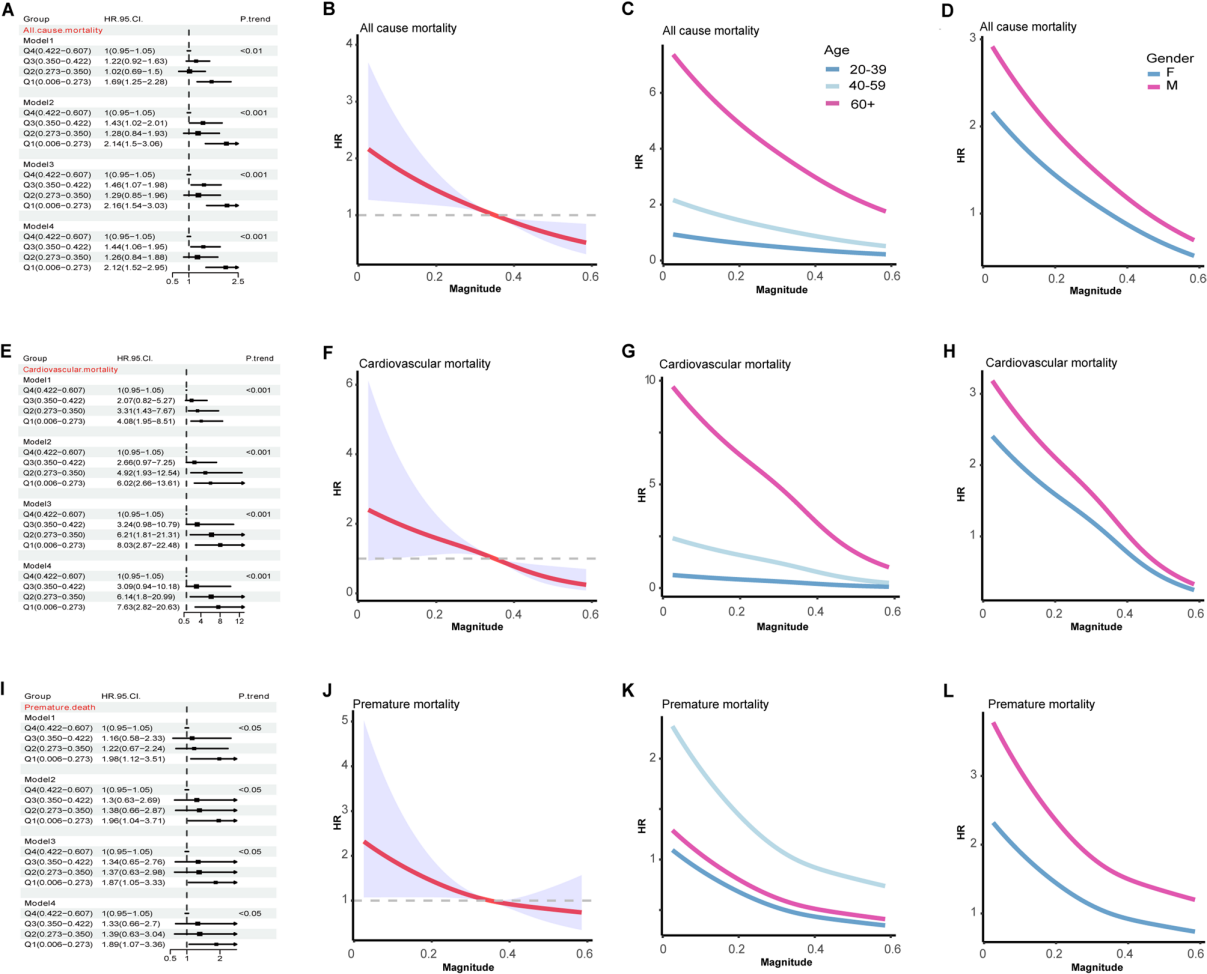
Association between phasor magnitude and mortality in overweight and obese adults. Panels **A**, **E** and **I** present forest plots of multivariable-adjusted hazard ratios (HRs) with 95% CIs across quartiles of phasor magnitude reference to Q4 for all‐cause mortality (**A**), cardiovascular mortality (**E**) and premature mortality (**I**) in four Cox models (Model 1–4). Panels **B**, **F** and **J** show restricted cubic spline curves of HR (solid red line) and 95% CI (shaded area) as a continuous function of phasor magnitude for the same outcomes. Panels **C**–**D**, **G**–**H** and **K**–**L** depict age‐stratified (20–39, 40–59, ≥ 60 years) and gender‐stratified (female, male) HR curves for all‐cause (**C**, **D**), cardiovascular (**G**, **H**) and premature (**K, L**) mortality

After establishing the association between phasor magnitude and the risk of advanced CKM syndrome, we next aimed to investigate its impact on the mortality. We find that lower phasor magnitude was significantly associated with increased risk of all-cause mortality across all models (Fig. 3A). In the fully adjusted Model 4, participants in the lowest quartile (Q1) had a more than two-fold higher mortality risk compared to the highest quartile (Q4), with a hazard ratio (HR) of 2.12 (95% CI: 1.52–2.95). An approximately inverse association between phasor magnitude and all-cause mortality was observed, as determined by restricted cubic spline analysis (Fig. 3B). In the age-stratified analysis (Fig.3C), the association was strongest among individuals aged 60 years and older, indicating that the mortality risk linked to circadian misalignment may be amplified in older adults compared to younger age groups. When stratified by sex (Fig.3D), this trend persisted in both males and females, with a steeper slope observed among males, suggesting greater sensitivity to circadian disruption. Further, we observed lower phasor magnitude quartiles were associated with a stepwise increase in cardiovascular mortality risk, with a HR of 7.63 (95% CI: 2.82–20.63) (Fig. 3E). A significant inverse association was observed between phasor magnitude and cardiovascular mortality (Fig. 3F). This association was strongest among individuals aged ≥ 60 years and was more pronounced in males than in females, as shown in age/sex-stratified analyses (Fig. 3G H). In addition, we examined premature death and found that lower phasor magnitude quartiles were associated with a stepwise increase in premature mortality risk across all models (Fig. 3I). Restricted cubic spline analysis demonstrated an inverse association between phasor magnitude and premature death that was consistent across age and gender subgroups (Fig. 3J–L). However, no statistically significant association was observed between phasor acrophase and adverse outcomes including all-cause mortality, cardiovascular mortality and premature death (Figure S3).

**Fig. 4:**
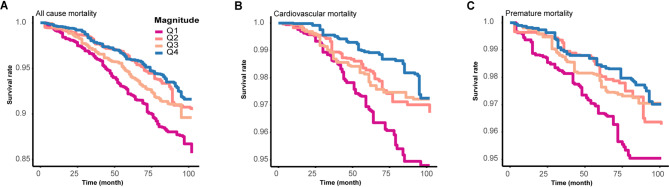
Predicted survival curves for mortality stratified by phasor magnitude quartiles. Survival was modeled using Cox proportional hazards regression with MORTSTAT (mortality status) as the outcome and phasor magnitude divided into quartiles (Q1-Q4, with Q1 lowest and Q4 highest). (**A**) All-cause mortality; (**B**) Cardiovascular mortality; (**C**) Premature death. Time on the x-axis is expressed in months since baseline; y-axis shows predicted survival probability

Kaplan–Meier survival curves further illustrated the prognostic significance of circadian alignment. Individuals in the lowest phasor magnitude quartile (Q1) exhibited the lowest survival probability for all-cause mortality over the 100-month follow-up, with a graded increase in survival observed across quartiles Q2 to Q4 (Fig. 4A). A similar pattern was observed for cardiovascular mortality (Fig. 4B), where lower phasor magnitude was associated with substantially reduced survival, and the separation between quartiles was more pronounced than for all-cause mortality. For premature mortality (Fig. 4C), survival probability also declined progressively with decreasing circadian amplitude, reinforcing the adverse impact of circadian misalignment across multiple mortality outcomes.

## Disscussion

Our study provides novel evidence that reduced circadian amplitude, quantified by phasor magnitude, is independently associated with the risk of advanced CKM syndrome and elevated mortality in overweight and obese adults. These associations persisted after rigorous adjustment for demographic, behavioral, and clinical covariates, indicating that circadian amplitude disruption contributes independently to CKM pathophysiology beyond established risk factors such as hypertension and diabetes. Demonstrating an inverse relationship between phasor magnitude and the risk of advanced CKM syndrome extends prior research on individual cardiometabolic outcomes to an integrated CKM framework and emphasizes rhythm robustness as a unifying marker of multi-organ dysfunction [21–23]. Our results align closely with and extend prior observational studies — linking shift-work–induced misalignment to 20–40% increases in all‐cause and cardiovascular mortality [24–26]—by specifically focusing on overweight and obese adults and demonstrating that lower phasor magnitude is associated with a > 2-fold higher risk of all-cause mortality, markedly increased cardiovascular mortality, and elevated premature death risk in fully adjusted models. Mechanistic insights support these associations: dampened circadian amplitude disrupts the synchronized regulation of glucose homeostasis, lipid metabolism, inflammatory responses, and blood pressure rhythms, thereby accelerating cardiovascular, renal, and metabolic pathologies and contributing to higher all-cause, cardiovascular, and premature mortality [27–29]. In contrast, phasor acrophase showed no significant relationships with CKM syndrome, all-cause mortality, cardiovascular mortality, or premature death. This discrepancy likely reflects fundamental differences in what these metrics represent. Phasor magnitude quantifies the strength of light–activity coupling, a marker of circadian alignment that is closely linked to cardiometabolic regulation. In contrast, phasor acrophase captures only the timing of the rhythm, and moderate shifts in timing may be buffered by behavioral or social factors, thereby exerting relatively limited impact on long-term clinical outcomes.

In subgroup analyses, the inverse association between phasor magnitude and the risk of advanced CKM appeared broadly consistent across age and sex strata, suggesting no strong evidence of effect modification. However, it is noteworthy that the overall risk levels were higher among older adults and in males. This pattern indicates that although the relative protective association of stronger circadian rhythmicity was similar across demographic groups, the absolute burden of CKM attributable to circadian disruption may be greater in these high-risk populations because of their elevated baseline risk [30–32]. Such a distinction underscores the importance of differentiating between relative effect consistency and population-level impact: circadian rhythmicity exerts a universal protective role, but its implications are amplified in groups with higher underlying susceptibility. Interestingly, the age-stratified analyses revealed heterogeneity across different mortality outcomes. While the protective associations of higher phasor magnitude with all-cause and cardiovascular mortality were more evident in older adults, the associations with premature death did not follow the same pattern. This discrepancy suggests that the adverse consequences of circadian disruption may manifest through distinct biological or behavioral pathways depending on the outcome of interest. For instance, the stronger links with all-cause and cardiovascular mortality in older adults may reflect cumulative vascular damage, systemic inflammation, and metabolic inflexibility that progressively accumulate with aging [33]. In contrast, premature mortality may be more strongly influenced by behavioral and environmental risk factors, such as shift work, lifestyle irregularities, or occupational exposures, which are not confined to older age groups. Interestingly, For all mortality-related outcomes assessed in this study, the protective associations of higher phasor magnitude were particularly evident in males. Sex-related influences may contribute: the relative cardiometabolic protection conferred by estrogen in premenopausal women contrasts with the combined effects of testosterone decline, higher visceral adiposity, and greater exposure to irregular sleep–wake schedules in men [34]. These mechanisms may explain why circadian misalignment exerts a disproportionate impact on mortality outcomes among older adults and males, even though its influence on CKM progression remains broadly consistent.

From a clinical perspective, objective assessment of circadian amplitude via wearable devices could improve CKM risk stratification beyond traditional measures [35]. However, current clinical assessment of circadian rhythm disruption remains suboptimal. While actigraphy offers convenient, noninvasive behavioral monitoring, it inadequately reflects endogenous circadian phase or amplitude. Biomarker-based measures, such as dim light melatonin onset or core body temperature, provide physiological precision but are resource-intensive and rarely feasible in routine practice. Subjective tools, including sleep diaries and questionnaires, are vulnerable to recall bias and may not adequately capture true circadian misalignment. Moving forward, integrating wearable biosensors, continuous physiological monitoring, and advanced computational modeling presents a promising avenue for achieving scalable, noninvasive, and accurate quantification of circadian alignment. In parallel with advancing measurement strategies, the evaluation of circadian amplitude–enhancing interventions is warranted. Interventions such as timed light exposure, structured sleep–wake schedules, and time-restricted feeding have been shown to strengthen circadian amplitude and improve cardiometabolic outcomes. Importantly, the subgroup patterns identified here may provide a rationale for precision prevention strategies in which circadian interventions are tailored to demographic and biological profiles.

Several limitations merit consideration. First, the observational design precludes causal inference, and the associations observed may be influenced by unmeasured confounders such as undiagnosed sleep disorders, psychosocial stressors, or genetic predispositions. Second, reverse causation cannot be excluded, as circadian disruption may arise secondary to CKM syndrome rather than contributing to its development. Additionally, it should be noted that the phasor-based assessment of circadian rhythms represents an indirect measure of circadian alignment, reflecting the overall synchronization between light exposure and activity patterns. However, this approach does not capture other dimensions of circadian behavior, such as individual chronotype or social jetlag, which may independently influence metabolic and cardiovascular outcomes. Beyond these methodological considerations, issues of generalizability and long-term inference also warrant attention. Although NHANES provides a representative sample of U.S. adults, generalizability to populations with different cultural or environmental circadian influences requires confirmation. Follow-up duration, while sufficient for initial mortality assessment, may not reflect long-term CKM trajectories. Future research should validate these associations in independent, diverse cohorts with repeated circadian assessments and detailed phenotyping to elucidate temporal relationships between amplitude changes and CKM progression.

## Conclusions

Our findings underscore reduced circadian amplitude as a novel, independent predictor of advanced CKM syndrome and adverse mortality outcomes in overweight and obese adults. Recognizing circadian robustness as a multi-organ health determinant supports further investigation of circadian-targeted interventions to reduce CKM-related morbidity and mortality.

## Supplementary Information

Below is the link to the electronic supplementary material.

Supplementary Material 1

Supplementary Material 2

## Data Availability

The datasets analyzed during the current study are publicly available from the U.S. Centers for Disease Control and Prevention NHANES repository. Derived analytic code and processed data can be obtained from the corresponding author upon reasonable request.

## Abbreviations

CKM: Cardio-kidney-metabolic
NHANES: National Health and Nutrition Examination Survey
CKD: Chronic kidney disease
OR: Odds ratio
HR: Hazard ratio
CI: Confidence interval
