# Supplementary material for: Circadian disruption is associated with advanced cardio-kidney-metabolic syndrome in overweight and obese adults: evidence from the NHANES database
Source: Nutr Metab (Lond). 2026 Jan 8;23:20. doi: 10.1186/s12986-025-01072-2 (PMC12882222; doi:10.1186/s12986-025-01072-2)
Supplement: Supplementary file 1 — Supplementary Material 1 [file 12986_2025_1072_MOESM1_ESM.docx]

**Supplementary Table 1： Baseline Demographic and Clinical Characteristics of the Study Population With Applying Survey Weights**

| **Characteristic** | **Overall N = 110,542,951^1^** | **1 N = 24,697,949^1^** | **2 N = 26,293,674^1^** | **3 N = 28,945,001^1^** | **4 N = 30,606,327^1^** | **p-value^2^** |
| --- | --- | --- | --- | --- | --- | --- |
| **Magnitude** | 0.36 (0.28.0.43) | 0.22 (0.17.0.25) | 0.32 (0.29.0.33) | 0.39 (0.37.0.40) | 0.47 (0.44.0.50) | <0.001 |
| **Age** | 50 (38.62) | 48 (34.59) | 48 (37.61) | 52 (41.64) | 53 (40.63) | <0.001 |
| **Gender = male** | 55,729,117 (50%) | 13,001,421 (53%) | 13,299,267 (51%) | 14,252,698 (49%) | 15,175,731 (50%) | 0.7 |
| **Race** |  |  |  |  |  | <0.001 |
| Mexican American | 10,142,261 (9.2%) | 1,606,959 (6.5%) | 2,349,889 (8.9%) | 2,642,871 (9.1%) | 3,542,543 (12%) |  |
| Hispanic | 6,609,450 (6.0%) | 1,322,740 (5.4%) | 1,660,571 (6.3%) | 1,861,290 (6.4%) | 1,764,849 (5.8%) |  |
| Non-Hispanic White | 75,177,471 (68%) | 15,694,662 (64%) | 16,901,195 (64%) | 20,133,124 (70%) | 22,448,490 (73%) |  |
| Non-Hispanic Black | 12,866,065 (12%) | 4,493,419 (18%) | 3,865,213 (15%) | 2,750,833 (9.5%) | 1,756,600 (5.7%) |  |
| Other | 5,747,704 (5.2%) | 1,580,168 (6.4%) | 1,516,807 (5.8%) | 1,556,882 (5.4%) | 1,093,846 (3.6%) |  |
| **Education** |  |  |  |  |  | <0.001 |
| College graduate or above | 31,103,348 (28%) | 6,934,901 (28%) | 9,053,486 (34%) | 8,528,429 (29%) | 6,586,532 (22%) |  |
| High school graduate | 24,145,890 (22%) | 5,338,793 (22%) | 4,948,981 (19%) | 5,707,374 (20%) | 8,150,742 (27%) |  |
| Less_than_high_school | 18,053,165 (16%) | 3,005,697 (12%) | 3,711,725 (14%) | 5,266,996 (18%) | 6,068,748 (20%) |  |
| Some college | 37,219,940 (34%) | 9,401,858 (38%) | 8,579,483 (33%) | 9,442,202 (33%) | 9,796,396 (32%) |  |
| Unknown | 20,609 | 16,700 | 0 | 0 | 3,909 |  |
| **Household.income** |  |  |  |  |  | 0.3 |
| <20,000 | 16,254,289 (15%) | 3,883,724 (16%) | 4,100,787 (16%) | 4,041,121 (14%) | 4,228,657 (14%) |  |
| 20,000–44,900 | 32,187,128 (30%) | 7,705,635 (32%) | 6,754,874 (26%) | 8,271,227 (30%) | 9,455,392 (32%) |  |
| 45,000–74,900 | 21,938,402 (20%) | 4,350,969 (18%) | 5,095,324 (20%) | 6,063,418 (22%) | 6,428,692 (22%) |  |
| 75,000+ | 37,147,375 (35%) | 8,178,963 (34%) | 9,586,855 (38%) | 9,610,054 (34%) | 9,771,505 (33%) |  |
| Unknown | 3,015,756 | 578,658 | 755,835 | 959,182 | 722,081 |  |
| **BMI** | 30.4 (27.5.34.5) | 30.8 (27.7.35.8) | 30.5 (27.4.34.7) | 30.2 (27.5.34.6) | 30.1 (27.4.33.4) | <0.001 |
| **Smoking** | 18,757,188 (17%) | 5,254,095 (21%) | 4,152,451 (16%) | 4,430,230 (15%) | 4,920,411 (16%) | 0.032 |
| **Alcoh.week** |  |  |  |  |  | <0.001 |
| <1/week | 38,863,562 (35%) | 9,409,951 (38%) | 9,390,423 (36%) | 9,907,836 (34%) | 10,155,352 (33%) |  |
| 1/day above | 4,988,291 (4.5%) | 447,944 (1.8%) | 908,953 (3.5%) | 1,311,663 (4.5%) | 2,319,731 (7.6%) |  |
| 1/week to <1/day | 30,931,362 (28%) | 6,581,318 (27%) | 7,573,578 (29%) | 8,107,189 (28%) | 8,669,277 (28%) |  |
| NO | 35,759,736 (32%) | 8,258,736 (33%) | 8,420,721 (32%) | 9,618,313 (33%) | 9,461,967 (31%) |  |
| **Hypertension** | 50,632,008 (46%) | 10,953,307 (44%) | 12,114,146 (46%) | 14,073,436 (49%) | 13,491,119 (44%) | 0.2 |
| **Diabetes** | 19,061,020 (17%) | 4,581,696 (19%) | 4,395,370 (17%) | 5,091,623 (18%) | 4,992,331 (16%) | 0.6 |
| **adv.CKM_stage** | 14,517,602 (15%) | 3,246,731 (15%) | 3,354,968 (14%) | 4,141,315 (16%) | 3,774,588 (14%) | 0.6 |
| **Albuminuria** |  |  |  |  |  | 0.2 |
| A1 | 89,006,389 (90%) | 19,248,463 (88%) | 21,756,320 (92%) | 23,306,263 (90%) | 24,695,343 (91%) |  |
| A2 | 8,211,885 (8.3%) | 2,243,552 (10%) | 1,613,924 (6.8%) | 2,176,086 (8.4%) | 2,178,323 (8.0%) |  |
| A3 | 1,377,875 (1.4%) | 415,247 (1.9%) | 344,042 (1.5%) | 279,486 (1.1%) | 339,100 (1.2%) |  |
| Unknown | 11,946,801 | 2,790,686 | 2,579,388 | 3,183,167 | 3,393,560 |  |
| **eGFR** |  |  |  |  |  | 0.037 |
| G1 | 60,631,709 (61%) | 13,433,733 (61%) | 14,288,074 (60%) | 14,898,567 (58%) | 18,011,335 (66%) |  |
| G2 | 32,108,934 (33%) | 7,095,510 (32%) | 8,169,733 (34%) | 8,863,187 (34%) | 7,980,504 (29%) |  |
| G3a | 3,998,178 (4.1%) | 807,980 (3.7%) | 790,271 (3.3%) | 1,517,219 (5.9%) | 882,708 (3.2%) |  |
| G3b | 1,386,487 (1.4%) | 374,502 (1.7%) | 398,163 (1.7%) | 413,582 (1.6%) | 200,240 (0.7%) |  |
| G4 | 408,121 (0.4%) | 160,556 (0.7%) | 56,905 (0.2%) | 63,283 (0.2%) | 127,376 (0.5%) |  |
| G5 | 92,096 (<0.1%) | 64,357 (0.3%) | 11,140 (<0.1%) | 5,996 (<0.1%) | 10,603 (<0.1%) |  |
| Unknown | 11,917,425 | 2,761,311 | 2,579,388 | 3,183,167 | 3,393,560 |  |
| **CKD.risk** |  |  |  |  |  | 0.028 |
| hig | 2,494,282 (2.5%) | 737,146 (3.4%) | 664,498 (2.8%) | 732,687 (2.8%) | 359,951 (1.3%) |  |
| low | 84,834,285 (86%) | 18,420,664 (84%) | 20,876,566 (88%) | 21,710,125 (84%) | 23,826,930 (88%) |  |
| mod | 10,160,497 (10%) | 2,387,166 (11%) | 1,919,591 (8.1%) | 3,110,588 (12%) | 2,743,152 (10%) |  |
| vhig | 1,136,461 (1.2%) | 391,662 (1.8%) | 253,631 (1.1%) | 208,435 (0.8%) | 282,733 (1.0%) |  |
| Unknown | 11,917,425 | 2,761,311 | 2,579,388 | 3,183,167 | 3,393,560 |  |
| **Heart failure** | 3,496,419 (3.2%) | 939,379 (3.8%) | 817,048 (3.1%) | 971,134 (3.4%) | 768,858 (2.5%) | 0.3 |
| ^1^ Median (Q1.Q4); n (%) | | | | | | |
| ^2^ Design-based KruskalWallis test; Pearson’s X^2: Rao & Scott adjustment | | | | | | |
